# Pharmacogenetic landscape of epidermal growth factor receptor mutations in Kurdish non-small cell lung cancer patients: implications for precision oncology

**DOI:** 10.3332/ecancer.2026.2087

**Published:** 2026-03-12

**Authors:** Badraldin K Hamad, Sami Saleem Omar, Iman H F Alnaqshabandy, Rawaz D Tawfeeq, Sarween A Abdullah, Aveen R Jalal

**Affiliations:** 1 Pharmacology Department, College of Pharmacy, Hawler Medical University, Erbil 44001, Iraq; 2 Preclinical Department, School of Medicine, University of Kurdistan Hewler, Erbil 44001, Iraq; 3 Pharmacology Department, College of Pharmacy, Knowledge University, Erbil 44001, Iraq; 4 Medical Oncologist, Rizgary Oncology Center - Rizgary Teaching Hospital, Erbil 44001, Iraq; 5 Faculty of Medicine, Koya University, Koya City, Erbil 44001, Iraq; 6 Department of Scientific Research Center, College of Science, University of Duhok, Duhok, Kurdistan Region 42001, Iraq; 7 Clinical Analysis Department, College of Pharmacy, Hawler Medical University, Erbil 44001, Iraq; 8 School of Sport, Exercise and Health Sciences. Loughborough University, 42001 Loughborough, UK

**Keywords:** EGFR mutations, Kurdish population, non-small cell lung cancer, precision medicine, tyrosine kinase inhibitors

## Abstract

This work presents the first thorough examination of epidermal growth factor receptor (EGFR) mutation prevalence in Kurdish patients with non-small cell lung cancer (NSCLC), identifying unique molecular and epidemiological traits. We found an overall EGFR mutation rate of 18.2% among 149 metastatic non-squamous NSCLC cases, which is comparable to Middle Eastern cohorts but much lower than rates reported in East Asian populations (47.2%–64.2%). Significant gender differences were found, with females (55.6%) having EGFR mutations at a significantly higher rate than males (44.4%, *p* = 0.0056). For Exon 19 deletions, this difference was especially noticeable (73.3% in females versus 66.7% in males). EGFR positivity was significantly higher in non-smokers (70.4%) than in smokers (29.6%, *p* < 0.0001), indicating a strong correlation between smoking status and mutation frequency. Significant variations in the distribution of mutations according to smoking status were found by subtype-specific analyses: L858R (4 versus 1 case, *p* = 0.04) and Exon 19 deletions (13 versus 6 cases, *p* = 0.001) were more common in non-smokers, while Exon 20 insertions showed no significant association (1 female case, *p* = 0.7147). According to available data, rare G719X/S768I variants (2.0%) may benefit from Afatinib, but Exon 19 deletions were the most common subtype in clinical settings (70.4% of EGFR-positive cases), indicating the use of first-line Osimertinib. By establishing that Kurdish NSCLC patients having a unique molecular subgroup, these findings highlight the significance of population-specific genomic profiling in precision oncology and cast doubt on long-held beliefs regarding the epidemiology of EGFR mutations in Asian populations. However, the study's retrospective design and small sample sizes for uncommon mutation subtypes are among its limitations, which call for additional prospective validation in larger cohorts. These findings highlight the need for specialised diagnostic and treatment approaches in underrepresented populations and advance our understanding of the heterogeneity of EGFR mutations across ethnic groups.

## Introduction

Lung cancer is one of the most prevalent and the leading cause of cancer-related deaths worldwide, with an estimated 1.8 million deaths every year [1, 2]. Lung cancer can be divided into two types, small cell lung cancer (SCLC) and non-SCLC (NSCLC), by histological methods. NSCLC is the most prevalent and accounts for approximately 80% of all cases [3, 4]. The incidence and prevalence of NSCLC are influenced by several risk factors, including endogenous exposures such as oestrogen and genetic alterations and exogenous exposures such as smoking, radon radiation, asbestos, coal and infections [5]. Tobacco is the predominant exogenous risk factor associated with lung cancer, contributing approximately up to 80% [6]. Traditionally, lung cancer predominates in males more than in females, and this could be due to the risk association with smoking that leads to increased cases among males. However, the progressive smoking intake among females has resulted in increased lung cancer incidence, presenting a significant threat to the public health system [7]. The remaining percentages of lung cancer cases are not attributed to smoking [4]. Epidermal growth factor receptors (EGFRs) are members of the ErbB family and play vital roles in normal physiological and cancerous conditions. The function of EGFR can be dysregulated in various types of cancers [8]. The EGFR mutation represents one of the most notable genetic alterations in NSCLC due to its implications in tumour growth, development and response in targeted therapy [9]. The therapeutic approach has been drastically changed by the identification of EGFR mutations and the subsequent development of EGFR tyrosine kinase inhibitors (TKIs), particularly in patients with the most prevalent mutations, such as exon 19 deletions and L858R point mutations in exon 21 [8, 10]. On the other hand, studies have shown that patients who harbour uncommon EGFR mutations exhibit different therapeutic responses [2, 11, 12]. Both prevalent and uncommon mutations are found in metastatic tumours in Asians, females, non-smokers and patients with lung adenocarcinoma. Moreover, studies show that the higher the mutation rate, the higher the survival rate and the better the therapeutic response [4–6]. However, further studies are required to unveil the mechanisms that underpin these outcomes. These types of mutations are more commonly observed among certain populations, including women, non-smokers and individuals of Asian descent [13, 14]. However, research on EGFR mutations in the Kurdish population remains scarce.

Three main goals are pursued by this study: (1) using comparative genomic analysis, create the first comprehensive EGFR mutation profile in Kurdish NSCLC patients; (2) look into smoking-related and gender-specific mutation patterns in this population; and (3) create customised treatment plans based on molecular epidemiology. The results will establish a precision medicine framework for EGFR-targeted treatment optimisation in Middle Eastern populations.

## Methods

This study has been carried out as an observational, retrospective, cross-sectional evaluation of EGFR mutation prevalence in stage IV metastatic non-squamous NSCLC amongst the Kurdish population. To show a representation of the Kurdish population, we have only taken samples from patients of Kurdish nationality from cancer treatment centres in the three major cities in the Kurdistan region, including Erbil, Sulaymaniyah and Duhok, and excluded other minorities. The samples were collected in the period between December 2022 to December 2024. A 149 patients were included in this study.

Tissue samples were obtained from these patients and formalin-fixed and paraffin-embedded (FFPE). To ensure proper preservation of molecular moieties, samples were processed and preserved in accordance with established guidelines [15]. Idylla™ EGFR Mutation Assay (Biocartis, Country) was used to analyse EGFR mutation in the samples with a fully automated real-time PCR system. This assay has the advantage of detecting EGFR mutations with great sensitivity and specificity in results that have been comparable to NGS [16, 17]. Using the Idylla™ EGFR Mutation Assay, we experimented on the FFPE samples directly with minimal sample preparation, and the results were obtained within 2–3 hours.

Demographic and clinical patient data were collected. The primary results were focused on the EGFR mutation status of the patients included in the study. The descriptive statistics method was used to state the patients’ characteristics and the rate of mutation occurrence. Ethical approval was granted by the Ministry of Health Duhok Directorate General of Health, and the University of Duhok for ethics under the approval NO. 13042025-33. A waiver of consent was requested from the ethics committee because of the retrospective and anonymised nature of the study. By generating accurate data on EGFR mutation, this study has been designed to help build a strategy for personalised treatment in Kurdish patients diagnosed with NSCLC.

Chi-square and Fisher’s exact test were carried out to determine statistical variances of EGFR mutations among men and women. A separate Chi-square analysis was performed to demonstrate the relation between EGFR mutations and smoking. Statistical analysis was conducted using the GraphPad Prism statistical package. Chi-square test was also used to determine variances in specific EGFR mutation in relation to gender and smoking.

## Result

### EGFR status and gender distribution

This comprehensive study investigates the prevalence of the EGFR mutations in correlation with gender, smoking status and specific mutation types. Two main statistical methods, chi-square and Fisher’s exact tests, were used to determine the statistical significance. A total of 149 patients with diagnosed stage 4 lung cancer were included in this study, consisting of 100 males and 49 females. Among them, 28 (18.79%) were tested EGFR positive, whereas 121 (81.2%) were EGFR negative. The majority of the EGFR-positive patients were female, with 57%, while male was 43%. On the other hand, the gender distribution in the EGFR-negative group was 88 males and 33 females (Table 1). The correlation between EGFR-positive and gender was assessed using Fisher's exact test. The results show a significant association between gender and EGFR status (*p* value = 0.0056).

### Smoking status and EGFR positivity

Smoking is one of the most significant risk factors that are observed in lung cancer. Among 149 patients, 100 were smokers and 49 were non-smokers. Smoking status among EGFR positives were as follows, 8 were smokers and 20 were non-smokers. However, results of the EGFR negative have shown the majority of patients were smokers, 92, while 29 of them were non-smokers (Table 1). To determine the associations between smoking status and EGFR positive, a chi-square test was performed. The statistical analysis exhibits a significant association between patients with EGFR positivity and non-smoker with a *p* value of 0.0001.

### EGFR mutations and gender

**Figure 1.**
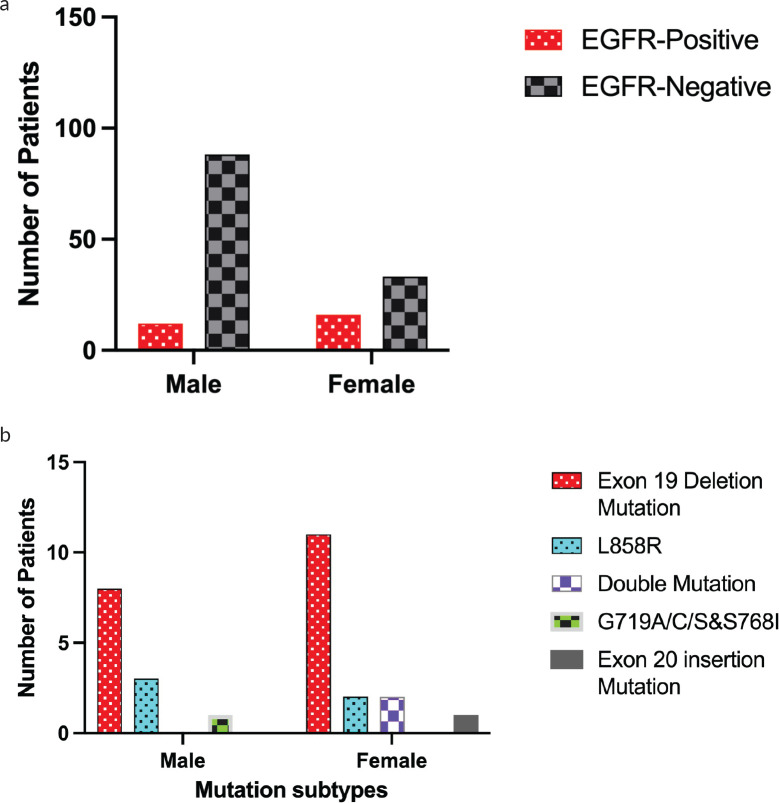
Sex-specific distribution of EGFR mutation in Kurdish lung cancer. (a) shows overall EGFR mutation frequency by sex, while (b) demonstrates mutation subtypes distribution by sex.

Several EGFR mutations have been investigated in this study, and their relationship with gender was also examined. These mutation types are distributed at the tyrosine kinase domain using Idylla^TM^ EGFR mutation test. It is a robust and rapid test that covers 51 mutations in exons 18, 19, 20 and 21. The study shows that the most prevalent mutation obtained was Exon 19 deletion (*n* = 19), which was observed in 11 females and 8 males, as shown in Table 2 and Figure 1. This association was assessed using a chi-square test that yielded a value of 6.171 (df = 1), with a *p* value of 0.0115. These results suggest a statistically significant association between gender and the presence of the Exon 19 deletion mutation. Thus, the mutation is more frequent in females compared to males (*p* < 0.05). However, Further studies are required to investigate potential biological or clinical implications of this gender-specific variation.

The second most common mutation identified is the L858R mutation, which is located in exon 21. The L858R mutation was identified in 3 males and 2 females. Based on the results obtained from a chi-square test with a value of 0.1186 (df = 1), and a *p* value of 0.7305, no statistically significant relationship between gender and the L858R mutation (*p* > 0.05) was observed, as shown in Table 2 and Figure 1. This suggests that there is no association of gender with the occurrence of the L858R mutation type in this study sample. Whereas, double mutations in which any two mutations of either Exon 19 deletion or L858R and T790M were observed in the patient, were identified in two female patients; however, it was absent in the male. Although the frequency of this type of mutation is low, the chi-square value of 4.137 (df = 1), with a *p* value of 0.1067 obtained from a chi-square test indicated a significant correlation between gender and the occurrence of double mutation Table 2. In this case, the occurrence of double mutation is higher in females than male patients. On the other hand, both the G719A/C/S and S768I mutations were observed in male (no = 1), while it was absent in females. The data obtained from the chi-square test indicates no significant correlation between gender and the occurrence of the G719A/C/S and S768I mutation type, with a value of 0.4933 (df = 1) and the *p* value 0.2546**.** There is no significant correlation between gender and the occurrence of Exon 20 insertion mutation type with a *p* value 0.1517.

### Association between EGFR mutations and smoking status

**Figure 2.**
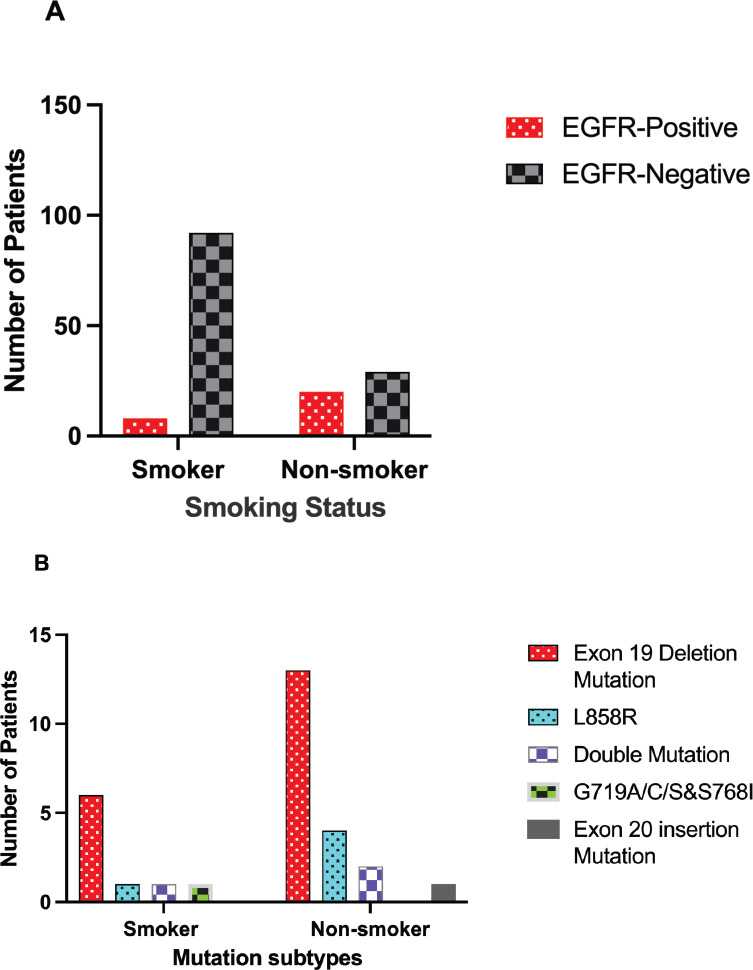
Impact of smoking status on EGFR mutation profiles in Kurdish lung cancer. (a): Overall EGFR positive by smoking status. (b): Exon 19 deletion frequency by smoking status.

To examine whether smoking history influenced mutation frequency, the distribution of EGFR mutations in relation to smoking status was analysed. The most common mutation among smokers was Exon 19 deletion that was detected in six cases, followed by only one case in all of the other mutation types, including L858R and G719A/C/S & S768I. In the non-smokers category, the Exon 19 deletion mutation was also the most frequently detected and was identified in 13 cases. Other mutations observed in non-smokers included L858R Mutation (four cases), Double Mutation (two cases) and Exon 20 deletion (one case), while the G719A/C/S & S768I mutation was not detected in this category Table 3 and Figure 2.

Although the Exon 19 deletion mutation was significantly more frequent in non-smokers compared to smokers, the difference was statistically significant (*p* = 0.001). These findings indicate that smoking status does significantly influence the occurrence of the Exon 19 deletion mutation, as shown in Table 3. Moreover, the L858R mutation exhibits more frequent in smoker than non-smoker category with statistically significant (*p* = 0.04), as shown in Table 3.

a b

## Discussion

The EGFR has a critical role in cell proliferation and survival pathways, with activating mutations frequently occurring in NSCLC [18]. The prevalence of EGFR mutation varies by geographical region and ethnicity, with the highest rate observed in Asia (38.4%), followed by North and South America (24.4%), and the lowest in Europe (14.1%) [14]. Building on this global context, our study provides the first thorough description of EGFR mutation patterns in Kurdish NSCLC patients, which also identifies gender differences that are clinically significant and suggests treatment options tailored to individual mutations.

Regarding therapeutic implications, although studies have shown that EGFR TKIs are effective in both populations [19], emerging evidence suggests non-Asian populations may derive greater benefit compared to Asian populations in both the adjuvant and metastatic settings, even though EGFR mutation rates are higher in non-Asian populations [20, 21]. Notably, the findings from our study demonstrate a markedly lower frequency of EGFR mutations (18.2%) among Kurdish patients with metastatic non-squamous NSCLC compared to the broader Asian populations reported in the PIONEER study [22]. Three important findings emerge from this analysis: (1) distinct gender disparities; (2) epidemiological differences from other Asian subgroups and (3) clinically actionable mutation patterns.

Focusing first on population differences, the EGFR mutation frequency in our cohort is substantially lower than that observed in East and Southeast Asian subgroups, which ranged from 47.2% to 64.2%, including populations from China, Taiwan, Vietnam and Thailand. Notably, even when compared to the Indian subgroup in the PIONEER study – which exhibited the lowest frequency among the Asian regions at 22.2% – the Kurdish population showed a slightly reduced prevalence. These data suggest that the Kurdish population, while geographically situated in Asia, may have a distinct molecular and genetic profile more closely aligned with that of White or Caucasian populations, where EGFR mutation frequencies are typically reported between 15% and 20% [14].

Turning to gender disparities, EGFR mutations are notably more common in females (55.6% of EGFR+ cases) than in Asian (usually 50%–55%) and Western (often <45%) populations [23]. This difference was especially noticeable for Exon 19 deletions, which were found to be 2.8 times more common in Kurdish women than in men (20.4% versus 9.0%). The extent of this effect in our cohort indicates that other ethnic-specific factors might be at play, even though oestrogen-mediated APOBEC3B activation has been suggested as a mechanism for female-predominant EGFR mutagenesis [23]. A distinct mutagenic microenvironment may be produced by the interaction between the CYP1A1*2A polymorphism, which is known to be common in Middle Eastern populations [24], and endogenous oestrogen metabolism. This underscores the importance of region-specific molecular profiling and highlights the need to avoid generalising mutation prevalence based on continental or broad ethnic classifications. Therefore, identifying both the overall mutation rate and specific EGFR point mutation subtypes within each racial or ethnic population is essential, given the observed differences in treatment response across these groups [20, 21]. In addition, the Kurdish cohort showed an EGFR mutation rate that is closely aligned with the MENA region's pooled average of 21.2% as reported in Benbrahim et al.'s meta-analysis [25]. This suggests that Kurdish patients share molecular characteristics with broader Middle Eastern populations, rather than East or Southeast Asians. The underlying mechanisms may involve hormonal influences, genetic predisposition or differential environmental exposures.

The observed smoking association patterns reveal complex relationships with mutation subtypes. Both the L858R (4 versus 1 cases, *p* = 0.04) and Exon 19 deletions (13 versus 6 cases, *p* = 0.001) mutation frequencies were significantly different between smokers and non-smokers, with non-smokers exhibiting a higher prevalence for both mutation types. There was only one instance of Exon 20 insertion mutations (in a female non-smoker), and there was no discernible variation by gender (*p* = 0.151) or smoking status (*p* = 0.7147). According to these findings, EGFR mutations in Kurdish patients might result from complex mechanisms other than conventional tobacco-associated carcinogenesis. To determine whether the exclusive presence of G719X/S768I mutations in male smokers (2.0% versus 0% in non-smokers, *p* = 0.32) is a population-specific pattern, further investigation is required.

This study shows that EGFR positivity is significantly higher in non-smokers, as shown in Table 1, than in smokers, which is consistent with well-established data indicating that EGFR mutations are more common in lung adenocarcinoma patients who have never smoked [26, 27]. When analysing specific EGFR mutation subtypes, we observed that the Exon 19 deletion was significantly more prevalent compared to other subtypes, which aligns with other studies [28] and we found a rare point mutation, G719A/C/S & S768I.

Given its demonstrated superiority in this subgroup (median PFS 21.4 versus 10.2 months versus first-generation TKIs), the third-generation TKI Osimertinib should be used as the first-line treatment for Exon 19 deletions, which account for 70.4% of EGFR+ cases [29]. Kurdish women may benefit especially from upfront Osimertinib, as indicated by the female predominance of these mutations. Our data support the use of Afatinib for G719X/S768I compounds, which have shown activity against these uncommon variants (ORR 77.8% in LUX-Lung series) [30, 31]. The fact that it only occurs in male smokers suggests that further mutation testing should be given priority for this subgroup.

Furthermore, when specific EGFR mutation subtypes were analysed, it was observed that Exon 19 deletion mutations were significantly more prevalent in females than males when considering the total study population (*n* = 142). In contrast, no significant difference in specific EGFR mutations between smokers and non-smokers. Treatment-naive patients showed a trend toward higher EGFR+ rates (66.7% versus 33.3% in pre-treated cases), emphasising the importance of early molecular testing before therapy initiation. The complete restriction of double mutations to female patients represents a novel finding without clear precedent in the literature. While the small sample size (*n* = 2) requires cautious interpretation, this observation aligns with emerging evidence of sex-specific differences in DNA repair efficiency [32].

In conclusion, although EGFR mutation is a well-established predictive biomarker in non-squamous NSCLC, both in the advanced and, more recently, the adjuvant setting, studies have highlighted variability in response rates across different racial and ethnic groups. This study represents the first investigation of EGFR mutation prevalence in the Kurdish population of Iraq, a developing country. Despite residing in the Asian continent – where EGFR mutation rates are generally high – the Kurdish population demonstrated one of the lowest mutation frequencies reported in Asian cohorts. These findings underscore the importance of further research to evaluate the therapeutic response and clinical efficacy of anti-EGFR therapies specifically within this population. Our research provides specific recommendations for clinical practice while challenging established epidemiological paradigms by identifying unique gender and molecular patterns of EGFR mutations in Kurdish NSCLC. These results support customised diagnostic and treatment algorithms for patients in the Middle East and highlight the significance of population-specific genomic research. A framework for precision therapy in this population is provided by the proven effectiveness of Osimertinib for common Exon 19 deletions and Afatinib for uncommon G719X/S768I variants. This study is constrained by its retrospective design, which inhibited comprehensive smoking history (pack-years) and multivariate analysis owing to the limited cohort of EGFR-positive cases; prospective validation in a more extensive population is essential to substantiate these findings.

## Conflicts of interest

The authors declare no conflicts of interest.

## Funding

No funds and grants were received.

## Figures and Tables

**Table 1.** Demographic and clinical characteristics based on distribution of Kurdish participant patients in Iraq who exhibited EGFR mutation.

Variables	EGFR - positive	EGFR - negative	Total	χ^2^	*p* value
Gender status					
Male	12 (42.85%)	88 (72.72%)	100	7.678	0.0056*
Female	16 (57.15%)	33 (27.27%)	49		
Smoking status					
Smokers	8 (28.57%)	92 (76.03%)	100	20.99	<0.0001*
Non-smokers	20 (71.43%)	29 (23.97%)	49		

EGFR: epidermal growth factor receptor. χ^2^: Chi-squared.

* *p* < 0.05

**Table 2.** The prevalence of EGFR subtype mutation based on gender.

Subtypes	Male (*n* = 100)	Female (*n* = 49)	*p* value
EXON 19 DELETION	8 (8%)	11 (22.44%)	0.0115*
L858R	3 (3%)	2 (4.08%)	0.7305
Double mutations	0 (0%)	2 (4.08%)	0.1067
G719A/C/S & S768I	1 (1%)	0 (0%)	0.2546
EXON 20 INSERTION	0 (0%)	1 (2.04%)	0.1517
Total in positive	12 (12%)	16 (32.65%)	

* *p* < 0.05

**Table 3.** The prevalence of EGFR subtype mutation based on smoking status.

Subtypes	Smokers (*n* = 100)	Non-smokers (*n* = 49)	*p* value
EXON 19 DELETION	6 (6%)	13 (26.53%)	0.001*
L858R	1 (1%)	4 (8.16%)	0.04*
Double mutations	0 (0%)	2 (4.08%)	0.1067
G719A/C/S & S768I	1 (1%)	0 (0%)	0.1
EXON 20 INSERTION	0 (0%)	1 (2.04%)	0.7147
Total in positive	8 (8%)	20 (40.82%)	

* *p* < 0.05
